# Mercury Biogeochemical Cycle in Yanwuping Hg Mine and Source Apportionment by Hg Isotopes

**DOI:** 10.3390/toxics11050456

**Published:** 2023-05-14

**Authors:** Xingang Jin, Junyao Yan, Muhammad Ubaid Ali, Qiuhua Li, Ping Li

**Affiliations:** 1 Key Laboratory for Information System of Mountainous Area and Protection of Ecological Environment of Guizhou Province, Guizhou Normal University, Guiyang 550001, China; jinxingang0725@foxmail.com; 2 State Key Laboratory of Environmental Geochemistry, Institute of Geochemistry, Chinese Academy of Sciences, Guiyang 550081, China; yanjunyao@mail.gyig.ac.cn (J.Y.); ubaid@mail.gyig.ac.cn (M.U.A.)

**Keywords:** mercury, mine wastes, surface water, paddy soil, Hg isotopes, source apportionment

## Abstract

Although mercury (Hg) mining activities in the Wanshan area have ceased, mine wastes remain the primary source of Hg pollution in the local environment. To prevent and control Hg pollution, it is crucial to estimate the contribution of Hg contamination from mine wastes. This study aimed to investigate Hg pollution in the mine wastes, river water, air, and paddy fields around the Yanwuping Mine and to quantify the pollution sources using the Hg isotopes approach. The Hg contamination at the study site was still severe, and the total Hg concentrations in the mine wastes ranged from 1.60 to 358 mg/kg. The binary mixing model showed that, concerning the relative contributions of the mine wastes to the river water, dissolved Hg and particulate Hg were 48.6% and 90.5%, respectively. The mine wastes directly contributed 89.3% to the river water Hg contamination, which was the main Hg pollution source in the surface water. The ternary mixing model showed that the contribution was highest from the river water to paddy soil and that the mean contribution was 46.3%. In addition to mine wastes, paddy soil is also impacted by domestic sources, with a boundary of 5.5 km to the river source. This study demonstrated that Hg isotopes can be used as an effective tool for tracing environmental Hg contamination in typical Hg-polluted areas.

## 1. Introduction

Mercury (Hg) is a highly toxic heavy metal that can travel a long distance in the atmosphere and is therefore considered a global pollutant [1]. The toxicity of Hg depends on its chemical form. The elevated levels of Hg in the air are mostly attributed to industrial emissions, such as coal burning, Hg mining, gold mining, wastes incinerators, and cement production [2]. Methylmercury (MeHg) is neurotoxic, and it has the ability to bioaccumulate and become ultimately biomagnified in the food web. Humans are exposed to MeHg mainly through the consumption of food [3,4,5]. The Minamata Convention went into effect in August 2017 to reduce the effects of Hg exposure on human health [6,7].

The Wanshan Hg Mine is considered the “capital of Hg” in China. Since 2002, mining activities have been banned at the site due to the depletion of Hg resources and the environmental implications [8,9]. However, long-term Hg mining activities have produced a large amount of mine wastes, which are an important source of Hg pollution in the surrounding atmosphere and surface water. Most Hg calcine piles are distributed at the source of the river. Under external forces, such as rainwater leaching, surface runoff, and wind erosion, the Hg from the mine wastes is released and enters the downstream water system [10,11,12]. Therefore, evaluating the ecological risks caused by Hg mines is crucial for local ecological restoration.

The mine wastes from Hg mines can diffuse into the surrounding environment through water and atmospheric transportation. The paddy soils are more heavily contaminated by Hg in Hg mining areas compared with other areas [13]. Among the crops grown in the Wanshan Hg mining area, rice has been identified as significantly capable of bioaccumulating MeHg in its grain, and rice ingestion could be the main route of MeHg exposure for local residents [14,15,16,17], which can pose serious health risks [18]. To avoid persistent paddy soil Hg pollution and subsequent human MeHg exposure, the sources of the paddy soil Hg need to be identified, and the Hg emissions from these sources can then be controlled by optimizing the major emission processes.

The Hg stable isotopes are an effective tool to track pollution sources and environmental processes [19,20,21,22]. There are seven natural stable isotopes of Hg: ^196^Hg, ^198^Hg, ^199^Hg, ^200^Hg, ^201^Hg, ^202^Hg, and ^204^Hg. Mercury isotopes not only have mass-dependent fractionation (MDF) (reported as δ^202^Hg), but they also have mass-independent fractionation (MIF, mainly reported as Δ^199^Hg or Δ^201^Hg). Mass-dependent fractionation can occur in physical, chemical, and biological processes, while MIF only occurs in a few specific processes, such as the photochemical reduction of Hg^2+^ and the photodegradation of MeHg [19,23,24,25,26]. Mixing models based on Hg isotopic MDF and MIF values has been used to quantify the contribution of the primary Hg sources in sediments [27,28,29]. Song et al. [6] used binary and ternary mixed models to calculate soil Hg pollution sources and their contribution ratios at different polluted sites. Yan et al. [30] used ternary mixed models to analyze the contribution ratios of the main Hg pollution sources in river water in Hg mining areas, and Fu et al. [31] used them for the quantitative source apportionment of Hg in the atmosphere. These studies indicate that the binary and ternary mixed models have been helpful in tracing the sources and biogeochemical processes of Hg in the environment. However, the contribution ratio of Hg mine wastes to the paddy soil in Hg mining areas remains unclear.

This study had the following aims: (1) to study the impact of the Hg mine wastes on the surrounding soil, water, and atmosphere; (2) to use Hg isotopic mixed models for the source apportionment of the Hg pollution in the downstream river water and paddy soil; and (3) to provide a theoretical basis for the source control of soil Hg pollution in paddy fields.

## 2. Materials and Methods

### 2.1. Study Area

**Figure 1:**
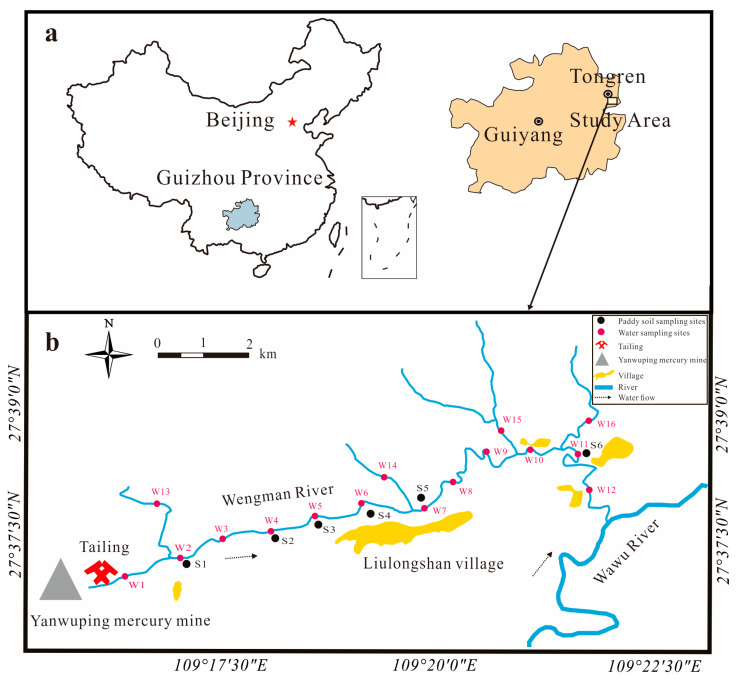
Location of study area (**a**) and distribution of sample sites (**b**).

The Wanshan Hg Mine is located in Guizhou Province, southwest China (Figure 1a). Mineralization at the Wanshan Hg Mine is primarily associated with thin-layered, laminated, fine-grained, dolomite or limestone beds of the mid-Cambrian age. The wall rocks are intensively altered by silicification, dolomitization, calcification, subordinate bituminization, and pyritization [32]. The primary ore mineral in the Hg deposits is cinnabar, with less metacinnabar [33].The Yanwuping Hg Mine (YMM) is one of the largest Hg mines in the Wanshan area. The Yanwuping Hg Mine is hilly and karstic, and it is located at an altitude of 340–1010 m. The climate is subtropical humid, with an annual rainfall of 1200–1400 mm and an annual temperature of 15 °C [34]. 

The Yanwuping Hg Mine’s historic Hg extraction facility and about 3.1 × 10^5^ m^3^ of mine wastes are located at the upper Wengman River [35]. In 2011, the government renovated the YMM and tailing dams, but 1.3 × 10^4^ m^2^ of the calcine deposits remained. The Wengman River (Figure 1b) originates in the YMM zone and belongs to the Yangtze River basin, which has an average summer depth of 1 m and is directly affected by upstream mine wastes [36].

Mine wastes, surface-layer soils, and deep-layer soils were collected at Yanwuping Mercury Mine. W1–16 and S1–6 were the water and paddy soils sampling sites, respectively.

The total gaseous Hg sampling sites were the same as those for mine wastes, soils, and paddy soils.

### 2.2. Sample Collection

Water and atmospheric samples were collected and monitored twice, in December 2021 and August 2022, due to the high seasonal variability of the various indicators in the river water and atmosphere. The interannual variability in the soil Hg is not significant. Thus, soil and mine wastes samples were collected only once, in December 2021. There are two main types of mine wastes: calcines, the residues of Hg ore after high-temperature calcination, and waste rock, which is lower-grade surrounding rock [37]. Because most of the site has been restored, a total of 75 samples were collected from the surface layer and below 30 cm, and the difference between the restored area and bare area was compared and evaluated. Among them, 42 samples comprised surface soil, calcines, and waste rock, and 33 samples comprised deep soil, calcines, and waste rock. During the same period, the soil samples from the paddy fields downstream of the YMM were collected. For each site, a final sample composed of 3–5 subsamples was collected using the diagonal sampling method (15 paddy soil samples; Figure 1b). The collected soil, calcine, and waste rock samples were kept in clean polyethylene bags, air-dried, ground, and passed through a 200-mesh sieve, followed by total Hg (THg) and THg isotopic analysis.

The YMM downstream rainwater and surface water of the Wengman River were sampled for unfiltered THg, filtered dissolved Hg (DHg), DHg isotopes, particulate Hg (PHg) isotopes, anions, and cations. The THg and DHg isotopes samples were acidified with ultrapure hydrochloric acid, the cation samples with distilled nitric acid, and the anion samples without acid. The water samples were sealed in double-layer polyethylene bags, sent back to the laboratory, protected from light, and stored in a refrigerator at 4 °C. The analytical tests were completed within 28 days.

The total gaseous Hg (TGM) concentrations at the YMM and downstream paddy field sites were monitored 48 times using a portable RA-915+ Zeeman Hg Analyzer (Lumex, Saint Petersburg, Russia). The Lumex instrument’s detection limit was 0.5 ng/m^3^. The instrument instantaneously displays the TGM concentrations per second, and each sampling point dataset represents an average monitoring time of at least 5 min in the field [37].

### 2.3. Analytical Methods

Approximately 0.1 g of the mine wastes and soil samples (dry weight) were digested with a mixture of HNO_3_ and HCl (v:v = 1:3) for 2 h in a water bath at 95 °C. BrCl was added to the samples, and they were stored for 24 h for the conversion of all forms of Hg to Hg^2+^, followed by the addition of acidic SnCl_2_ to the solution to reduce the Hg ions to Hg^0^. They were analyzed using cold-vapor atomic absorption spectrometry (CVAAS, F732-S, Shanghai Huaguang Instrument Factory, Shanghai, China). The detection limit of this method was 0.1 μg/L.

To determine the concentrations of THg and DHg in a water sample, BrCl was added to the sample and allowed to oxidize for 24 h. The Hg ions in the solution were then reduced to Hg^0^ using acidic SnCl_2_. The samples were preconcentrated into gold tubes and were later tested using a cold-vapor atomic fluorescence spectrophotometer (CVAFS, Tekran 2500, Tekran, Toronto, Ontario, Canada). The detection limit of this method was 0.1 μg/L. The THg in the water passing through a 0.45 μm filter is defined as DHg; subtracting the DHg from the THg yields the concentration of PHg in the water [34]. The anions and cations were analyzed by automated Dionex ICS-90 ion chromatography (Dionex, Sunnyvale, CA, USA) and an inductively coupled plasma optical emission spectrometer (ICP-OES, Varian, Palo Alto, CA, USA), respectively [38].

The Hg isotopic composition was analyzed using Neptune Plus MC-ICP-MS (Thermo Fisher Scientific, Waltham, MA, USA)at the State Key Laboratory of Environmental Geochemistry, the Institute of Geochemistry, the Chinese Academy of Sciences, following the method described by Yin et al. [39]. The total soluble Hg (TSHg) of the Hg mine wastes was extracted using a leaching experiment, as the Hg isotopes were to be tested along with the digested soil sample [37]. To ensure the minimum Hg concentration required for the DHg isotopes analysis of aqueous samples, each filtered water sample was pre-enriched into 5 mL of 40.0% aqua regia absorbent solution (v:v, HNO_3_: HCl = 2:1), as shown in the method established by Li et al. [40]. For the Hg isotopes of PHg in the water samples, 2–5 L of water was filtered through a high-temperature purified Teflon membrane and freeze-dried. The Hg in the membrane was extracted into 5 mL of 40.0% anti-aqua regia absorbent solution using a tubular muffle furnace [41].

### 2.4. Hg Isotopes Analysis

The Hg isotopic composition was calculated using the formula presented by Blum and Bergquist (2007). Mass-dependent fractionation is expressed as delta (δ), and the results were calculated as follows:

δ^xxx^Hg_sample_ (‰) = [(^xxx/198^Hg_sample_/^xxx/198^Hg_NIST3133_ − 1)] × 1000 (1)

where xxx is 199, 200, 201, 202, or 204. Mass-independent fractionation is expressed as “Δ”, and it was calculated using the following equations:

Δ^199^Hg = δ^199^Hg − δ^202^Hg × 0.252 (2)

Δ^200^Hg = δ^200^Hg − δ^202^Hg × 0.502 (3)

Δ^201^Hg = δ^201^Hg − δ^202^Hg × 0.752 (4)

In this study, the binary mixed model was used to calculate the two sources of DHg of River Water No. 1. The calculations were performed using Equations (5) and (6) [1,42,43]:

δ^202^Hg_3_ = δ^202^Hg_1_ × F_1_ + δ^202^Hg_2_ × F_2_ (5)

1= F_1_ + F_2_ (6)

where F represents the percentage of the pollution source, subscript 1 represents the TSHg, subscript 2 represents the mountain spring water DHg, and subscript 3 represents the River Water No. 1 DHg. When calculating the two sources of River Water No. 1 PHg using a binary mixing model, subscript 1 represents the Hg mine wastes, subscript 2 represents the mountain spring water PHg, and subscript 3 represents the River Water No. 1 PHg.

The fractions of Hg in the paddy soil were derived from rainwater sources, river water sources, and geological background sources, and they were calculated using a triple-member mixing model as follows:

δ^202^Hg_soil_ = δ^202^Hg_rain_ × F_rain_ + δ^202^Hg_river_ × F_river_ + δ^202^Hg_nat_ × F_nat_ (7)

Δ^199^Hg_soil_ = Δ^199^Hg_rain_ × F_rain_ + Δ^199^Hg_river_ × F_river_ + Δ^199^Hg_nat_ × F_nat_ (8)

1 = F_rain_ + F_river_ + F_nat_ (9)

where the subscripts rain, river, and nat represent rainwater sources, river water sources, and geological background sources, respectively, and F_rain_, F_river_, and F_nat_ represent the percentages of rainwater sources, river water sources, and geological background sources, respectively.

### 2.5. Quality Control

The quality control included blanks, duplicate samples, and certified reference materials (CRMs). The mean THg concentration in the method blanks was 0.026 ng/mL. Duplicate samples were measured after every 10 samples, and the mean relative standard deviations of the THg in the duplicate samples were 3.00% (*n* = 16). The low, medium, and high concentrations of the soil and mine wastes samples were controlled using GSS-5 (soil, 0.290 mg/kg), CRM021 (soil, 5.00 mg/kg), and CC580 (sediment, 132 mg/kg), with mean recovery ratios of 99.3% ± 3.25%, 91.7% ± 1.06%, and 99.3% ± 5.22%, respectively. The mean recovery ratio for the enrichment experiment of the water sample was 101% ± 4.31%, and the mean recovery ratio of the CRM in the PHg concentration experiment of the water sample was 99.0% ± 8.40%. The results of the UM-Almadén standard solution (δ^202^Hg: −0.52‰ ± 0.05‰; Δ^199^Hg: 0.00‰ ± 0.04‰; Δ^201^Hg: −0.02‰ ± 0.04‰, *n* = 7) and the CC580 for sediment (δ^202^Hg: −0.47‰ ± 0.04‰; Δ^199^Hg: −0.06‰ ± 0.02‰; Δ^201^Hg: −0.04‰ ± 0.02‰, *n* = 3) were consistent with previous studies (CC580, δ^202^Hg: −0.51‰ ± 0.04‰; Δ^199^Hg: 0.00‰ ± 0.03‰; Δ^201^Hg: −0.02‰ ± 0.05‰, *n* = 2 [43,44,45,46]).

### 2.6. Data Analysis

Statistical analysis of the data, including means, standard deviations, and t-tests, was performed using IBM SPSS Statistics 26.0 (IBM, Armonk, NY, USA) and Microsoft Excel 2019 software (Microsoft, Redmond, WA, USA) (statistical significance = *p* < 0.05). Origin 2021 (OriginLab, Northampton, MA, USA) was used for the graphical demonstration of the data, and Arcmap 10.7 (ESRI, RedLands, CA, USA) was used to plot the spatial distributions by inverse distance weighting.

## 3. Results and Discussion

### 3.1. Hg Pollution in Mine Wastes

Considerable variation was observed in the THg concentrations of the YMM mine wastes. Except for the highest Hg concentration of 1.98 × 10^4^ mg/kg found in surface mine wastes, the THg concentrations in the remaining samples showed a geometric mean of 38.4 mg/kg, with a range of 1.60–358 mg/kg. For the deep mine wastes, the THg concentrations showed a geometric mean of 46.8 mg/kg, with a range of 14.5–1.07 × 10^3^ mg/kg. The considerable variations in the calcine THg concentrations may be attributed to the different retort furnaces used at the YMM. As the early smelting methods were not so advanced, inadequate ore burning resulted in low Hg recovery and a high concentration of calcines. The advancement in smelting technology led to adequate ore roasting, increased Hg recovery (≥95.0%), and a lower Hg concentration in the calcines [47]. The THg concentrations in the deep mine wastes analyzed in this study were higher than that of the local bedrock (0.35 mg/kg) [32], which was similar to the previous study also conducted at the Wanshan Hg Mine (geometric mean of THg concentrations: 49.0 mg/kg; THg concentration range: 4.15–825 mg/kg) [33]. The THg concentrations in 38.5% (15/39) of the YMM mine wastes samples exceeded the second-type soil pollution risk screening value (38.0 mg/kg) [48], and for 17.9% (7/39) of the samples, the THg concentrations exceeded the soil pollution risk control value of the second category of construction land (82.0 mg/kg) [48]. This demonstrates that a significant proportion of Hg persists even after the high-temperature melting of Hg ore [29].

**Figure 2:**
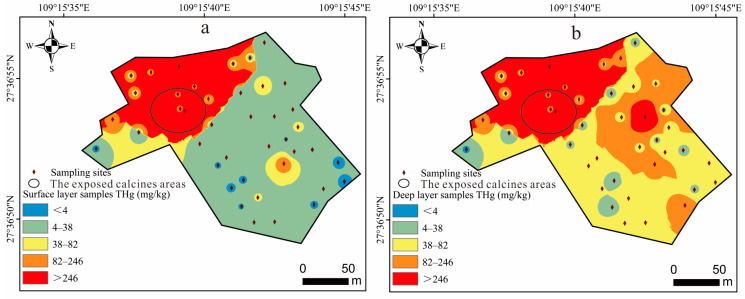
Spatial distribution of Hg pollution in mine wastes and soils at YMM: (**a**) surface layer; (**b**) deep layer.

The THg concentrations in the soils covered by restoration showed a geometric mean of 7.70 mg/kg, with a range of 1.68–139 mg/kg. The THg concentrations in the surface samples and deep samples correlated significantly (*p* < 0.05), indicating that the surface soil has been polluted by calcines in the lower layer. The soil THg concentrations were much higher than the agricultural land soil pollution risk control value (4.00 mg/kg, 6.5 < pH ≤ 7.5) [49], which is 70 times higher than the Guizhou Province soil background value of 0.110 mg/kg [50]. A comparison of the calcine area before and after the YMM restoration is shown in Figure 2a,b. The distribution of the Hg pollution in the surface and deep layers reveals that the most serious Hg pollution occurs in the exposed calcines areas. The mine wastes in the YMM are still the primary source of Hg pollution in the surrounding ecosystem. The exposed calcines seriously impact the local ecological environment by continually releasing Hg into the atmosphere, entering surface water bodies, and leaching into downstream farming soil [8,33].

### 3.2. Atmospheric Hg

**Figure 3:**
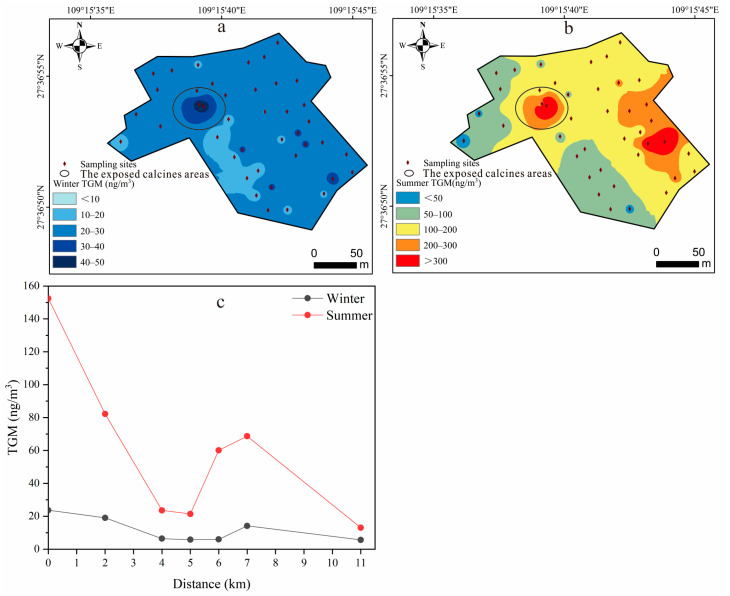
Spatial distribution of TGM at YMM and downstream paddy field: (**a**) spatial distribution of TGM at YMM in winter; (**b**) spatial distribution of TGM at YMM in summer; (**c**) variation in TGM with distance of paddy field downstream of YMM.

The spatial distribution of the TGM at the YMM showed significant variations (Figure 3a,b). In wintertime, the TGM concentrations averaged 24.1 ± 6.90 ng/m^3^, with a range of 10.1–45.0 ng/m^3^. In summertime, the TGM concentrations averaged 153 ± 129 ng/m^3^, with a range of 43.3–700 ng/m^3^. The TGM concentrations at the exposed calcines areas were found to be the highest both in winter and summer, and the TGM concentrations in summer were much higher than those in winter (winter: 45.0 ng/m^3^; summer: 700 ng/m^3^), while the TGM concentrations at the restored sampling point were much lower (winter: 23.6 ng/m^3^; summer: 153 ng/m^3^). The results show that mine wastes are still an important emission source of atmospheric Hg pollution, and remediated measures could effectively reduce the Hg emission flux at the interface between the mine wastes and air [51]. Compared with other Hg mining areas in China, the TGM concentrations in the YMM (43.3–700 ng/m^3^) were much higher than those in the Xunyang (7.40–410 ng/m^3^) and Wanshan (13.5–309 ng/m^3^) Hg mining areas [33,52]. However, the concentrations were much lower than those in the Xiushan Hg mining area (29.0–4.21 × 10^4^ ng/m^3^) [53]. The mean concentration of TGM was three times higher when compared with the air quality reference standard of 50.0 ng/m^3^ set by the Ministry of Environmental Protection of China, and it may pose a potential risk to local residents [54]. Therefore, the Hg emissions from mine wastes should be strictly controlled to reduce the environmental risks.

Previous studies have reported that the TGM released from pollution sources can settle into paddy soil after migration [55,56]. After the TGM monitoring in the downstream paddy fields, the following trends were noticed (Figure 3c). The results indicate that the TGM concentrations gradually decreased within 5.5 km of the YMM both in winter and summer (winter: 5.79 ng/m^3^; summer: 21.4 ng/m^3^). However, the TGM concentrations were still higher than the global background (1.50–1.60 ng/m^3^) [33,57]. The TGM concentrations considerably increased at around 5.5 km, with the highest value of 68.7 ng/m^3^ in summer, and then gradually decreased with the increasing distance. The increase in the TGM in the ambient air far from the YMM indicates the influence of nearby domestic Hg emission sources, such as Hg emissions from coal burning, waste incineration, traffic pollution, etc. [35,58,59].

### 3.3. Surface Water Hg and Source Apportionment

#### 3.3.1. Surface Water Hg Pollution

**Figure 4:**
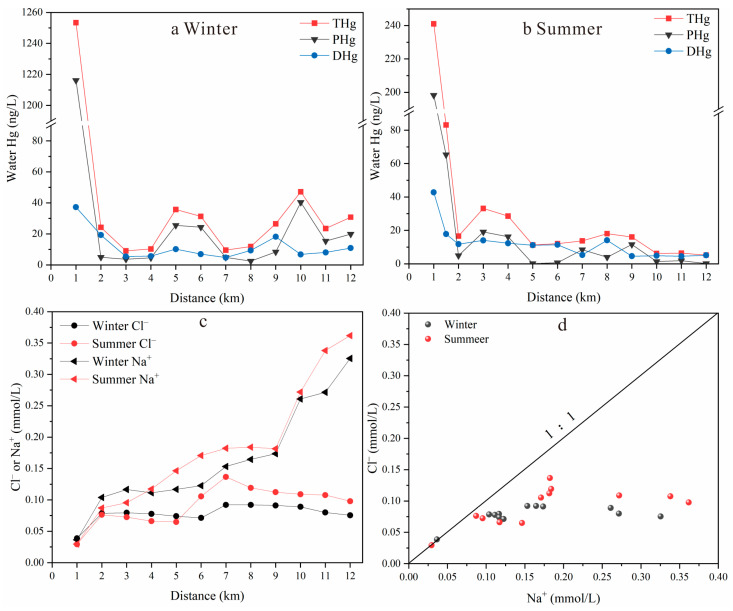
Water Hg and ion concentrations at Wengman River: (**a**) variation in different forms of Hg concentration with distance from YMM in winter; (**b**) variation in different forms of Hg concentration with distance from YMM in summer; (**c**) variation in Cl^−^, Na^+^ concentrations with distance from YMM; (**d**) evolution of Cl^−^/ Na^+^ ratios.

The Hg concentrations in the surface water of the Wengman River flowing through the YMM varied greatly. The THg, PHg, and DHg concentrations in the winter water samples averaged 101 ng/L (5.34–1.25 × 10^3^ ng/L), 86.9 ng/L (2.22–1.22 × 10^3^ ng/L), and 14.9 ng/L (3.12–80.9 ng/L), respectively. In the summer water samples, the averages were 34.4 ng/L (2.14–241 ng/L) for THg, 22.9 ng/L (0.08–198 ng/L) for PHg, and 11.5 ng/L (1.75–42.7 ng/L) for DHg. The Hg mine wastes upstream are considered to be the main source of the surface water Hg pollution [37,60]. The highest Hg concentration was found at the W1 site near the YMM (Figure 4a,b). The THg concentration in the winter surface water at this site exceeded the standard limit of 1000 ng/L stipulated by China’s Class V surface water environmental quality standard [61], indicating the direct influence of the Hg mine wastes. In order to reduce the impact of the upstream calcine leachate on the downstream water system, Xu et al. [36] designed and built a weir 1.5 km away from the YMM, which can intercept 40.4% of the THg per year and significantly reduce the THg concentrations in the river. The upstream calcine leachate is mainly composed of PHg. In this study, the proportions of the water PHg to THg in the winter and summer flowing through the weir decreased by 76.5% and 52.9%, respectively, indicating that the removal ratios were higher than those presented by Xu et al. [36]. The difference between the two studies might be attributed to the flow of the river water, as previous studies have reported that water flow is the main factor that influences the transport and migration of Hg [53,62]. In this study, the average THg concentrations in the summer samples (34.4 ng/L) were lower than those of the winter samples (101 ng/L). This might be because the summer samples were collected after a heavy rain event and the erosive, and leaching effect of the rainwater was not significant. The river flows in summer were higher than those in winter, which mainly showed that the dilution effect resulted in lower Hg concentrations in the summer samples. However, the highest concentration still exceeded the threshold limit of 100 ng/L set by China’s Class III surface water environmental quality standard [61].

The above studies have proven that the weir can indeed cause the particulate matter to settle, which is because the water flow slows down and the suspension time increases, thereby reducing the Hg pollution downstream. However, Xu et al. [36] only monitored before and after the weir, and they did not set sampling points downstream of the Wengman River. The results of this study show that at 4–5 km downstream of the Wengman River, the THg concentrations decreased to 10.3–11.3 ng/L, as a large amount of PHg had settled. The proportion of DHg to THg increased to 55.4%–98.9%, indicating the PHg sedimentation effect. The Hg concentrations at 4–5 km were close to the mean concentration of 7.09 ng/L in the tributaries, which indicated the baseline concentration of the surface water in this area. Except for W14, the water Hg concentrations in the tributaries in this study were similar to those of Qiu et al. [35] (tributary: 3.00–17.0 ng/L). After 5.5 km, the Hg concentration in the river water gradually increased, but it did not exceed the limit of 100 ng/L stipulated by China’s Class III surface water environmental quality standard [61]. The Hg concentration at 6.5 km of the tributary (W14 sample) in wintertime reached 85.0 ng/L, exceeding the 50.0 ng/L limited stipulated by China’s Class II surface water environmental quality standard [61]. This indicated that the surface water after 5.5 km may be impacted by other external sources of Hg.

This study analyzed the anions and cations of the Wengman River, and it found that the Na^+^ and Cl^−^ concentrations increased significantly after 5.5 km in both winter and summer. The increase in the Na^+^ concentration was much higher than that of Cl^−^ (Figure 4c). Figure 4d shows that the ratio of Cl^−^:Na^+^ in the sample at 1 km was 1:1, indicating that it was mainly derived from the dissolution of evaporite rock. However, the ratio of the water Cl^−^:Na^+^ downstream gradually fell below the 1:1 ratio line. The ratio of Cl^−^ and Na^+^ ranged from 0.232 to 0.876, which indicates the contribution of sources other than the dissolution of evaporite rocks [63,64]. Because Liulongshan Township is 5.5 km away, with a dense population, domestic activities have significant impacts on the water chemistry [65]. Cl^−^ is not affected by physical, chemical, or biological processes, and it is a good indicator of anthropogenic activities, such as the use of agricultural fertilizers, animal manure, and domestic sewage [66]. The Wanshan area belongs to the karst landform, and the main rock type is carbonate rock. The Na^+^ concentration in the surface water is relatively low. The increase in Na^+^ relative to Cl^−^ may be due to silicate weathering (e.g., plagioclase) and the effect of the input from domestic pollution sources. In order to further elucidate the contribution of both to the excess Na^+^, this study used the molar ratio bivariate plots of the Na^+^-normalized Ca^2+^ and Mg^2+^ and Na^+^-normalized Ca^2+^ and HCO_3_^−^ distributions (Figure S1) to identify the contribution of rock weathering to the ion source in the river [67,68]. As shown in Figure S1, the ionic composition of the river water in the study area was mainly located near the weathered end element of the carbonate rock, with a trend towards the weathered end element of the silicate rock. This indicates that the contribution of silicate rocks to river water ions is small. Therefore, this study concluded that the increase in Na^+^ concentration relative to the Cl^−^ concentration after 5.5 km from the Wengman River may be caused by domestic pollution [69,70]. This domestic pollution may come from domestic sewage, domestic waste (such as batteries, thermometers, pigment and paint residues, fluorescent lamps, and so on), etc. [59,71].

#### 3.3.2. Source Apportionment by Hg Isotopes

The river water THg increased significantly when it flowed through the Hg mining area (Figure 4a,b), which is consistent with previous literature [9,72]. In winter, the δ^202^Hg and Δ^199^Hg values in the river water samples downstream averaged −0.29‰ ± 0.30‰ (−0.71~0.11‰, *n* = 7) and −0.02‰ ± 0.07‰ (−0.12~0.06‰, *n* = 7), respectively (Table S1). Mercury isotopes were used to trace the source of Hg in the upstream water of the Wengman River. The results showed that the main contributing sources included Hg mine wastes and mountain spring water. This study assumes that the Hg from mine wastes and mountain spring water enters the River Water No. 1 sampling site in a rapid mixing process and that no significant MDF and MIF will occur during this process. The pollution sources of the DHg in the River Water No. 1 sample mainly include the TSHg from the Hg mine wastes and the DHg from the mountain spring water. For the TSHg of the Hg mine wastes, the DHg of the mountain spring water, and the DHg of River Water No. 1, the δ^202^Hg values were −0.90‰, −1.57‰, and −1.25‰, respectively, and the Δ^199^Hg values were all close to zero. Many previous studies have demonstrated the usefulness of end-member mixing models for Hg-source tracking in water environments [73,74]. In this study, the relative contributions of the different sources for the River Water No. 1 DHg were calculated using a binary mixing model. The relative contribution ratio of the mine waste TSHg to DHg was 48.6%, and that of the mountain spring water was 51.4%.

The pollution sources of the PHg in River Water No. 1 mainly included the Hg mine wastes and mountain spring water. The δ^202^Hg values in the Hg mine wastes, the PHg of mountain spring water, and the River Water No. 1 PHg were −0.35‰, −1.78‰, and −0.48‰, respectively, and the Δ^199^Hg values were all close to zero. The observed Δ^199^Hg values in Hg mine wastes are consistent with previous studies [29,75,76]. In this study, the relative contributions of the two pollution sources to the River Water No. 1 PHg were calculated using a binary mixing model. The relative contribution ratio of the Hg mine wastes was 90.5%, and that of the mountain spring water was 9.50%.

The THg in the River Water No. 1 was 1.25 × 10^3^ ng/L, while DHg accounted for 3.00%, and PHg for 97.0%. It was calculated that the Hg mine wastes directly contributed 89.3% to the river Hg pollution at the No. 1 site, indicating that the erosion of Hg mine wastes by runoff is the main process of the Hg pollution in the rivers near the Hg mine. This shows that the upstream water of the Wengman River is seriously polluted with Hg. The government needs to remediate the mine wastes left at the site and reinforce the tailings dam, which could reduce the Hg pollution in the downstream river and the health risk to local residents.

### 3.4. Paddy Soil Hg and Source Apportionment

#### 3.4.1. Paddy Soil Hg Pollution

The THg concentrations in the paddy soil downstream of the YMM averaged 3.58 ± 1.82 mg/kg with a range of 1.49–8.51 mg/kg. Compared with other Hg mining areas, the paddy soil THg concentrations at the YMM were lower than those in China’s Xunyang (1.30–750 mg/kg), Xiushan (0.45–68.0 mg/kg), and Wanshan (0.50–188 mg/kg) Hg mining areas [52,53,77]. However, they were still much higher than the agricultural soil pollution risk screening value (0.60 mg/kg, 6.5 < pH ≤ 7.5) and Guizhou soil Hg background value (0.110 mg/kg) [49,50]. The results showed that the downstream paddy soils are still seriously polluted by THg and indicated serious ecological risks. It is critical to identify the sources and contributions of Hg pollution in paddy soil. Therefore, preventive measures should be taken to control the Hg release. The obtained results provide a reliable theoretical and scientific basis for the treatment and safe utilization of Hg-contaminated soil.

The trends of the soil THg in the downstream paddy fields in this study were not consistent with those presented by Xu et al. [53], who reported that, with the increase in the distance from the Hg mining area, the soil THg concentrations tended to decrease. However, in this study, a different trend was noticed. A decreasing trend was observed prior to the 5.5 km distance; however, after 5.5 km, the THg concentrations at sites S4 and S5 significantly increased. The variation in the THg concentration in the downstream paddy soil is consistent with that of the TGM (Figure S2). Apart from this, a significant correlation was found between the TGM and paddy soil THg (*p* < 0.05). This indicates that atmospheric dry and wet depositions play a vital role in the Hg pollution of paddy soil [35]. The paddy fields are located along the banks of the Wengman River, and the local people have been using the Hg-contaminated river water for irrigation for a long time, which could also be a key source of the Hg pollution in the paddy soil [18,78].

#### 3.4.2. Source Apportionment by Hg Isotopes

**Figure 5:**
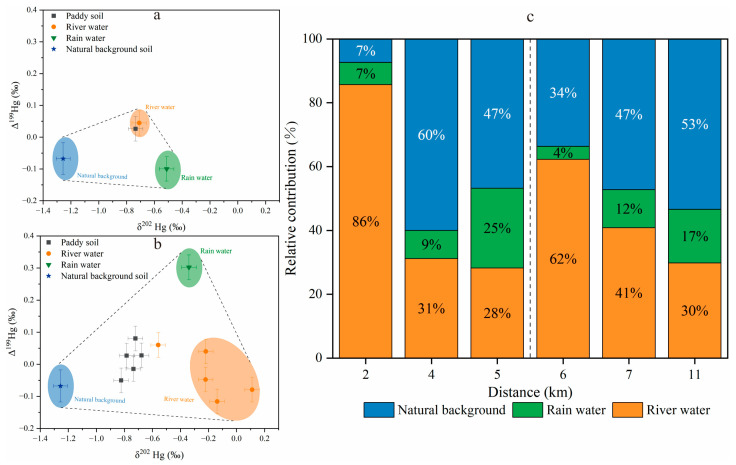
δ^202^Hg and Δ^199^Hg values from different sources and relative contributions to paddy soil: (**a**) soil Hg pollution source analysis before 2 km; (**b**) soil Hg pollution source analysis after 2 km; (**c**) three pollution sources of Hg in paddy soil. Natural background soil Hg isotopes values were adopted from Song et al. [6].

Previous studies have shown that soils can preserve the isotopic fingerprints of Hg pollution sources [42,79,80]. The paddy field downstream of the YMM are located on both sides of the Wengman River. There was a significant correlation between the TGM and THg in the paddy soil (*p* < 0.05), indicating that dry and wet atmospheric depositions are an important source of Hg pollution in the paddy soil. The contribution of wet deposition is of key importance as shown in Figure 5a,b. Pribil et al. [47] stated that the Hg in the soils to the north and east of the mining area may be the result of atmospheric deposition, geological background influence, and gaseous Hg emissions from calcines piles during Hg processing. The irrigation of paddy fields with Hg-contaminated river water is also one of the key sources [18].

The Rain Water No. 1 sample was collected 2 km away from the mining area. Due to its proximity to the Hg mining area, the Hg mine wastes have a greater impact. As shown in Figure 5a, the Hg isotopes compositions were δ^202^Hg = −0.51‰ ± 0.05‰ and Δ^199^Hg = −0.10‰ ± 0.04‰. The Rain Water No. 2 sample was collected at a distance of 6 km from the mining area, and we noticed that the Hg mine wastes had less of an impact. The Δ^199^Hg in the rainwater was positive, which was similar to the Hg isotopic value of the rainwater in Guiyang (δ^202^Hg: −0.44~−4.27‰, Δ^199^Hg: 0.19~1.16‰) [81]. The δ^202^Hg (δ^202^Hg = −0.34‰ ± 0.05‰) and Δ^199^Hg (Δ^199^Hg = 0.30‰ ± 0.04‰) values in the rainwaters are presented in Figure 5b. The mean values of the δ^202^Hg and Δ^199^Hg in the paddy soil were −0.73‰ ± 0.11‰ (−0.91~−0.56‰, *n* = 12) and 0.03‰ ± 0.05‰ (−0.05~0.10‰, *n* = 12), respectively. According to Song et al. [6], the mean values of the δ^202^Hg and Δ^199^Hg in the paddy soils in the Wanshan area were −1.26‰ ± 0.06‰ (−1.30~−1.21‰, *n* = 2) and −0.07‰ ± 0.10‰ (−0.14~0.00‰, *n* = 2), which were used as the background values in this study (Table S2).

As shown in Figure 5a,b, the combined characteristics of the Δ^199^Hg and δ^202^Hg indicate that the paddy soil is a ternary mixture of different sources, such as rainwater, river water, and geological background sources. Therefore, the δ^202^Hg and Δ^199^Hg of the corresponding point samples were used to trace the source of the Hg pollution in the paddy soil. This study calculated the relative contributions of the three sources using a ternary mixed model, as shown in Figure 5c. The results showed that the exogenous input of the Hg pollution in the paddy soil can be divided into two parts. The first part is the area contaminated by Hg mining activities. Within 5.5 km of the YMM, the river water is mainly polluted by Hg mine wastes, while the paddy soil pollution at 2 km is mostly attributed to river water, reaching 86.0%. The contribution range of river water was 28.0~86.0%, while in the case of rainwater, the range was 7.00~25.0%. The contribution range of the geological background was 7.00~60.0%. With the increase in distance, the Hg contribution of the river water gradually decreased, while the contribution ratio of the rainwater and geological background sources gradually increased. The paddy fields in this range were mainly contaminated by the Hg mine. The second part is the domestic Hg-polluted area. After 5.5 km from the YMM, the contribution of the river water to the paddy soil increased to 62.0% at 6 km, which is consistent with the increase in the Hg concentrations in the river water, indicating the influence of domestic pollution sources. The contribution ranges were 30.0~62.0% by river water, 4.00~17.0% by rainwater, and 34.0~53.0% by geological background. With the increase in distance, the contribution ratio of the river water gradually decreased, and the contribution ratio of the rainwater and geological background sources increased again, indicating that the paddy soil pollution in this range was mainly attributed to domestic pollution sources.

## 4. Conclusions

The YMM is still the primary source of Hg pollution in the surrounding ecosystem, and especially of the significant atmospheric Hg emissions in the exposed calcines area. Peak concentrations of THg were observed at the river upstream, and the source apportionment for the DHg and PHg in the river water using a binary mixing model demonstrated that mine wastes were the main source of Hg in the surface water. The TGM concentrations downstream showed specific spatial distributional characteristics, indicating a large amount of Hg emissions from Hg mine wastes and unidentified domestic pollution sources. This study calculated the contributions of the river water, atmospheric wet deposition, and geological background to the paddy soil Hg pollution by Hg isotopes, which were also verified by the spatial distributions of the river water Hg, river water anions and cations, TGM, and paddy soil Hg. The study shows that the paddy soil upstream at 5.5 km is mainly polluted by the Hg mine, while domestic sources are the main contributors after a distance of 5.5 km. This study provides an important scientific basis for the source control of Hg in the surface water and paddy fields in Hg mining areas. This is needed to control the Hg emissions from mine wastes to the river water and atmosphere, which would finally reduce the Hg bioaccumulation in agricultural crops and the associated human health risks in Hg-polluted areas.

## Data Availability

Not applicable.

## Supplementary Materials

The following supporting information can be downloaded at: https://www.mdpi.com/article/10.3390/toxics11050456/s1, Figure S1: Molar ratio bivariate plots of (a) Na+-normalized Ca2+ and Mg2+ and (b) Na+-normalized Ca2+ and HCO3–; Figure S2: Variation of THg with distance in paddy field downstream of YMM; Table S1: Mercury isotopes composition of water samples; Table S2: Mercury isotopic composition in soil samples.

## References

[1] Feng X., Foucher D., Hintelmann H., Yan H., He T., Qiu G. (2010). Tracing mercury contamination sources in sediments using mercury isotope compositions. Environ. Sci. Technol..

[2] Li P., Du B., Maurice L., Laffont L., Lagane C., Point D., Sonke J.E., Yin R., Lin C.-J., Feng X. (2017). Mercury isotope signatures of methylmercury in rice samples from the Wanshan mercury mining area, China: Environmental implications. Environ. Sci. Technol..

[3] Wright L.P., Zhang L., Cheng I., Aherne J., Wentworth G.R. (2018). Impacts and effects indicators of atmospheric deposition of major pollutants to various ecosystems—A review. Aerosol Air Qual. Res..

[4] Donovan P.M., Blum J.D., Singer M.B., Marvin-DiPasquale M., Tsui M.T.K. (2016). Isotopic Composition of Inorganic Mercury and Methylmercury Downstream of a Historical Gold Mining Region. Environ. Sci. Technol..

[5] Gerson J.R., Topp S.N., Vega C.M., Gardner J.R., Yang X., Fernandez L.E., Bernhardt E.S., Pavelsky T.M. (2020). Artificial lake expansion amplifies mercury pollution from gold mining. Sci. Adv..

[6] Song Z., Wang C., Ding L., Chen M., Hu Y., Li P., Zhang L., Feng X. (2021). Soil mercury pollution caused by typical anthropogenic sources in China: Evidence from stable mercury isotope measurement and receptor model analysis. J. Clean. Prod..

[7] Brocza F.M., Biester H., Richard J.-H., Kraemer S.M., Wiederhold J.G. (2019). Mercury Isotope Fractionation in the Subsurface of a Hg(II) Chloride-Contaminated Industrial Legacy Site. Environ. Sci. Technol..

[8] Li P., Feng X., Shang L., Qiu G., Meng B., Liang P., Zhang H. (2008). Mercury pollution from artisanal mercury mining in Tongren, Guizhou, China. Appl. Geochem..

[9] Qiu G.L., Feng X.B., Wang S.F., Fu X.W., Shang L.H. (2009). Mercury distribution and speciation in water and fish from abandoned Hg mines in Wanshan, Guizhou province, China. Sci. Total Environ..

[10] Kim K.-H., Kabir E., Jahan S.A. (2016). A review on the distribution of Hg in the environment and its human health impacts. J. Hazard. Mater..

[11] Li P., Feng X., Qiu G., Shang L., Li G. (2009). Human hair mercury levels in the Wanshan mercury mining area, Guizhou Province, China. Environ. Geochem. Health.

[12] Zhang C., Qiu G.L., Anderson C.W.N., Zhang H., Meng B., Liang L., Feng X.B. (2015). Effect of Atmospheric Mercury Deposition on Selenium Accumulation in Rice (*Oryza sativa* L.) at a Mercury Mining Region in Southwestern China. Environ. Sci. Technol..

[13] Xia J.C., Wang J.X., Zhang L.M., Anderson C.W.N., Wang X., Zhang H., Dai Z.H., Feng X.B. (2020). Screening of native low mercury accumulation crops in a mercury—Polluted mining region: Agricultural planning to manage mercury risk in farming communities. J. Clean. Prod..

[14] Feng X., Li P., Qiu G., Wang S., Li G., Shang L., Meng B., Jiang H., Bai W., Li Z. (2008). Human exposure to methylmercury through rice intake in mercury mining areas, Guizhou Province, China. Environ. Sci. Technol..

[15] Dai Z., Feng X., Sommar J., Li P., Fu X. (2012). Spatial distribution of mercury deposition fluxes in Wanshan Hg mining area, Guizhou province, China. Atmos. Chem. Phys..

[16] Du B., Feng X., Li P., Yin R., Yu B., Sonke J.E., Guinot B., Anderson C.W., Maurice L. (2018). Use of mercury isotopes to quantify mercury exposure sources in inland populations, China. Environ. Sci. Technol..

[17] Xing Y., Wang J., Xia J., Liu Z., Zhang Y., Du Y., Wei W. (2019). A pilot study on using biochars as sustainable amendments to inhibit rice uptake of Hg from a historically polluted soil in a Karst region of China. Ecotoxicol. Environ. Saf..

[18] Zhang Y., Zhou X., Ma W., Yin D., Wang Y., Zhang C., Wang D. (2022). Distribution of Mercury and Methylmercury in Farmland Soils Affected by Manganese Mining and Smelting Activities. Int. J. Environ. Res. Public Health.

[19] Kwon S.Y., Blum J.D., Yin R., Tsui M.T.-K., Yang Y.H., Choi J.W. (2020). Mercury stable isotopes for monitoring the effectiveness of the Minamata Convention on Mercury. Earth Sci. Rev..

[20] Jimenez-Moreno M., Barre J.P.G., Perrot V., Berail S., Rodriguez Martin-Doimeadios R.C., Amouroux D. (2016). Sources and fate of mercury pollution in Almaden mining district (Spain): Evidences from mercury isotopic compositions in sediments and lichens. Chemosphere.

[21] Wiederhold J.G., Smith R.S., Siebner H., Jew A.D., Brown G.E., Bourdon B., Kretzschmar R. (2013). Mercury Isotope Signatures as Tracers for Hg Cycling at the New Idria Hg Mine. Environ. Sci. Technol..

[22] Stetson S.J., Gray J.E., Wanty R.B., Macalady D.L. (2009). Isotopic Variability of Mercury in Ore, Mine-Waste Calcine, and Leachates of Mine-Waste Calcine from Areas Mined for Mercury. Environ. Sci. Technol..

[23] Bergquist B.A., Blum J.D. (2007). Mass-dependent and-independent fractionation of Hg isotopes by photoreduction in aquatic systems. Science.

[24] Blum J.D., Sherman L.S., Johnson M.W. (2014). Mercury isotopes in earth and environmental sciences. Annu. Rev. Earth Planet. Sci..

[25] Zhang H., Feng X., Larssen T., Qiu G., Vogt R.D. (2010). In inland China, rice, rather than fish, is the major pathway for methylmercury exposure. Environ. Health Perspect..

[26] Smith R.S., Wiederhold J.G., Jew A.D., Brown G.E., Bourdon B., Kretzschmar R. (2015). Stable Hg Isotope Signatures in Creek Sediments Impacted by a Former Hg Mine. Environ. Sci. Technol..

[27] Liu J., Feng X., Yin R., Zhu W., Li Z. (2011). Mercury distributions and mercury isotope signatures in sediments of Dongjiang, the Pearl River Delta, China. Chem. Geol..

[28] Yin R., Feng X., Chen B., Zhang J., Wang W., Li X. (2015). Identifying the Sources and Processes of Mercury in Subtropical Estuarine and Ocean Sediments Using Hg Isotopic Composition. Environ. Sci. Technol..

[29] Yin R., Feng X., Wang J., Li P., Liu J., Zhang Y., Chen J., Zheng L., Hu T. (2013). Mercury speciation and mercury isotope fractionation during ore roasting process and their implication to source identification of downstream sediment in the Wanshan mercury mining area, SW China. Chem. Geol..

[30] Yan J., Li R., Ali M.U., Wang C., Wang B., Jin X., Shao M., Li P., Zhang L., Feng X. (2023). Mercury migration to surface water from remediated mine waste and impacts of rainfall in a karst area—Evidence from Hg isotopes. Water Res..

[31] Fu X., Marusczak N., Wang X., Gheusi F., Sonke J.E. (2016). Isotopic Composition of Gaseous Elemental Mercury in the Free Troposphere of the Pic du Midi Observatory, France. Environ. Sci. Technol..

[32] Hua Y., Cui M. (1994). Wanshan Mercury Deposit in Guizhou Province.

[33] Yan J., Wang C., Wang Z., Yang S., Li P. (2019). Mercury concentration and speciation in mine wastes in Tongren mercury mining area, southwest China and environmental effects. Appl. Geochem..

[34] Zhang H., Feng X., Zhu J., Sapkota A., Meng B., Yao H., Qin H., Larssen T. (2012). Selenium in soil inhibits mercury uptake and translocation in rice (*Oryza sativa* L.). Environ. Sci. Technol..

[35] Qiu G., Feng X., Meng B., Zhang C., Gu C., Du B., Lin Y. (2013). Environmental geochemistry of an abandoned mercury mine in Yanwuping, Guizhou Province, China. Environ. Res..

[36] Xu X., Gu C., Feng X., Qiu G., Shang L., Xu Z., Lu Q., Xiao D., Wang H., Lin Y. (2019). Weir building: A potential cost-effective method for reducing mercury leaching from abandoned mining tailings. Sci. Total Environ..

[37] Li P., Feng X., Qiu G., Zhang J., Meng B., Wang J. (2013). Mercury speciation and mobility in mine wastes from mercury mines in China. Environ. Sci. Pollut. Res..

[38] Shao M., Liu Z., Sun H., Lai C., Ma Z., He X., Fang Y., Chai Q. (2023). C-N-P driven changes to phytoplankton community structure and gross primary productivity in river-fed reservoir ecosystems on the Chinese Loess Plateau. J. Hydrol..

[39] Yin R., Krabbenhoft D.P., Bergquist B.A., Zheng W., Lepak R.F., Hurley J.P. (2016). Effects of mercury and thallium concentrations on high precision determination of mercury isotopic composition by Neptune Plus multiple collector inductively coupled plasma mass spectrometry. J. Anal. At. Spectrom..

[40] Li K., Lin C.-J., Yuan W., Sun G., Fu X., Feng X. (2019). An improved method for recovering and preconcentrating mercury in natural water samples for stable isotope analysis. J. Anal. At. Spectrom..

[41] Fu X., Feng X., Yin R., Zhang H. (2013). Diurnal variations of total mercury, reactive mercury, and dissolved gaseous mercury concentrations and water/air mercury flux in warm and cold seasons from freshwaters of southwestern China. Environ. Toxicol. Chem..

[42] Estrade N., Carignan J., Donard O.F. (2011). Tracing and quantifying anthropogenic mercury sources in soils of northern France using isotopic signatures. Environ. Sci. Technol..

[43] Qin C., Du B., Yin R., Meng B., Fu X., Li P., Zhang L., Feng X. (2020). Isotopic Fractionation and Source Appointment of Methylmercury and Inorganic Mercury in a Paddy Ecosystem. Environ. Sci. Technol..

[44] Blum J.D., Bergquist B.A. (2007). Reporting of variations in the natural isotopic composition of mercury. Anal. Bioanal. Chem..

[45] Estrade N., Carignan J., Sonke J.E., Donard O.F. (2010). Measuring Hg isotopes in bio-geo-environmental reference materials. Geostand. Geoanal. Res..

[46] Janssen S.E., Johnson M.W., Blum J.D., Barkay T., Reinfelder J.R. (2015). Separation of monomethylmercury from estuarine sediments for mercury isotope analysis. Chem. Geol..

[47] Pribil M.J., Rimondi V., Costagliola P., Lattanzi P., Rutherford D.L. (2020). Assessing mercury distribution using isotopic fractionation of mercury processes and sources adjacent and downstream of a legacy mine district in Tuscany, Italy. Appl. Geochem..

[48] Ministry of Ecology and Environment of China (MEE) (2018). Soil Environmental Qualityrisk Control Standard for Soil Contamination of Development Land (GB3600–2018).

[49] Ministry of Ecology and Environment of China (MEE) (2018). Soil Environmental Quality Risk Control Standard for Soil Contamination of Agricultural Land (GB15618–2018).

[50] China National Environmental Monitoring Center (CNEMC) (1990). The Soil Environmental Background Value in the People’s Republic of China.

[51] Dai Z., Feng X., Zhang C., Wang J., Jiang T., Xiao H., Li Y., Wang X., Qiu G. (2013). Assessing anthropogenic sources of mercury in soil in Wanshan Hg mining area, Guizhou, China. Environ. Sci. Pollut. Res..

[52] Qiu G., Feng X., Meng B., Sommar J., Gu C. (2012). Environmental geochemistry of an active Hg mine in Xunyang, Shaanxi Province, China. Appl. Geochem..

[53] Xu X., Lin Y., Meng B., Feng X., Xu Z., Jiang Y., Zhong W., Hu Y., Qiu G. (2018). The impact of an abandoned mercury mine on the environment in the Xiushan region, Chongqing, southwestern China. Appl. Geochem..

[54] Ministry of Environment Protection of China (MEP) (2012). Ambient Air Quality Standards (GB3095–2012).

[55] Xu X., Liu N., Landis M.S., Feng X., Qiu G. (2016). Characteristics and distributions of atmospheric mercury emitted from anthropogenic sources in Guiyang, southwestern China. Acta Geochim..

[56] Zhao L., Qiu G., Anderson C.W., Meng B., Wang D., Shang L., Yan H., Feng X. (2016). Mercury methylation in rice paddies and its possible controlling factors in the Hg mining area, Guizhou province, Southwest China. Environ. Pollut..

[57] Fu X., Zhang H., Yu B., Wang X., Lin C.-J., Feng X. (2015). Observations of atmospheric mercury in China: A critical review. Atmos. Chem. Phys..

[58] Li Z.G., Feng X., Li P., Liang L., Tang S.L., Wang S.F., Fu X.W., Qiu G.L., Shang L.H. (2010). Emissions of air-borne mercury from five municipal solid waste landfills in Guiyang and Wuhan, China. Atmos. Chem. Phys..

[59] Das R., Wang X., Khezri B., Webster R.D., Sikdar P.K., Datta S. (2016). Mercury isotopes of atmospheric particle bound mercury for source apportionment study in urban Kolkata, India. Elem. Sci. Anthr..

[60] Zhang G., Liu C.-Q., Wu P., Yang Y. (2004). The geochemical characteristics of mine-waste calcines and runoff from the Wanshan mercury mine, Guizhou, China. Appl. Geochem..

[61] Environmental Protection Administration of China (EPA) (2002). Chinese Standards for Surface Water Quality (GB3838–2002).

[62] Qiu G., Feng X., Wang S., Xiao T. (2006). Mercury contaminations from historic mining to water, soil and vegetation in Lanmuchang, Guizhou, southwestern China. Sci. Total Environ..

[63] Yokoo Y., Nakano T., Nishikawa M., Quan H. (2004). Mineralogical variation of Sr–Nd isotopic and elemental compositions in loess and desert sand from the central Loess Plateau in China as a provenance tracer of wet and dry deposition in the northwestern Pacific. Chem. Geol..

[64] Zhang F., Jin Z., Yu J., Zhou Y., Zhou L. (2015). Hydrogeochemical processes between surface and groundwaters on the northeastern Chinese Loess Plateau: Implications for water chemistry and environmental evolutions in semi-arid regions. J. Geochem. Explor..

[65] Xu Z., Liu C.-Q. (2010). Water geochemistry of the Xijiang basin rivers, South China: Chemical weathering and CO_2_ consumption. Appl. Geochem..

[66] Yin C., Yang H., Wang J., Guo J., Tang X., Chen J. (2020). Combined use of stable nitrogen and oxygen isotopes to constrain the nitrate sources in a karst lake. Agric. Ecosyst. Environ..

[67] Gaillardet J., Dupre B., Allegre C.J., Negrel P. (1997). Chemical and physical denudation in the Amazon River basin. Chem. Geol..

[68] Gaillardet J., Dupre B., Louvat P., Allegre C.J. (1999). Global silicate weathering and CO_2_ consumption rates deduced from the chemistry of large rivers. Chem. Geol..

[69] Li X., Liu C., Harue M., Li S., Liu X. (2010). The use of environmental isotopic (C, Sr, S) and hydrochemical tracers to characterize anthropogenic effects on karst groundwater quality: A case study of the Shuicheng Basin, SW China. Appl. Geochem..

[70] Liu C., Li S., Lang Y., Xiao H. (2006). Using δ15N-and δ18O-values to identify nitrate sources in karst ground water, Guiyang, Southwest China. Environ. Sci. Technol..

[71] Mao Y., Cheng L., Ma B., Cai Y. (2016). The fate of mercury in municipal wastewater treatment plants in China: Significance and implications for environmental cycling. J. Hazard. Mater..

[72] Zhang H., Feng X.B., Larssen T., Shang L.H., Vogt R.D., Lin Y., Li P., Zhang H.I. (2010). Fractionation, distribution and transport of mercury in rivers and tributaries around Wanshan Hg mining district, Guizhou Province, Southwestern China: Part 2-Methylmercury. Appl. Geochem..

[73] Washburn S.J., Blum J.D., Kurz A.Y., Pizzuto J.E. (2018). Spatial and temporal variation in the isotopic composition of mercury in the South River, VA. Chem. Geol..

[74] Wiederhold J.G., Skyllberg U., Drott A., Jiskra M., Jonsson S., Bjorn E., Bourdon B., Kretzschmar R. (2015). Mercury isotope signatures in contaminated sediments as a tracer for local industrial pollution sources. Environ. Sci. Technol..

[75] Gehrke G.E., Blum J.D., Marvin-DiPasquale M. (2011). Sources of mercury to San Francisco Bay surface sediment as revealed by mercury stable isotopes. Geochim. Cosmochim. Acta.

[76] Sonke J.E., Schäfer J., Chmeleff J., Audry S., Blanc G., Dupré B. (2010). Sedimentary mercury stable isotope records of atmospheric and riverine pollution from two major European heavy metal refineries. Chem. Geol..

[77] Yin D., He T., Yin R., Zeng L. (2018). Effects of soil properties on production and bioaccumulation of methylmercury in rice paddies at a mercury mining area, China. J. Environ. Sci..

[78] Du J., Liu F., Zhao L., Liu C., Fu Z., Teng Y. (2021). Mercury horizontal spatial distribution in paddy field and accumulation of mercury in rice as well as their influencing factors in a typical mining area of Tongren City, Guizhou, China. J. Environ. Health Sci. Eng..

[79] Feng X., Yin R., Yu B., Du B. (2013). Mercury isotope variations in surface soils in different contaminated areas in Guizhou Province, China. Chin. Sci. Bull..

[80] Zhang H., Yin R.-S., Feng X.-B., Sommar J., Anderson C.W., Sapkota A., Fu X.-W., Larssen T. (2013). Atmospheric mercury inputs in montane soils increase with elevation: Evidence from mercury isotope signatures. Sci. Rep..

[81] Wang Z.H., Chen J.B., Feng X.B., Hintelmann H., Yuan S.L., Cai H.M., Huang Q., Wang S.X., Wang F.Y. (2015). Mass-dependent and mass-independent fractionation of mercury isotopes in precipitation from Guiyang, SW China. Comptes Rendus Geosci..

