# Supplementary material for: Mercury Biogeochemical Cycle in Yanwuping Hg Mine and Source Apportionment by Hg Isotopes
Source: Toxics. 2023 May 14;11(5):456. doi: 10.3390/toxics11050456 (PMC10222634; doi:10.3390/toxics11050456)
Supplement: Supplementary file 1 [file toxics-11-00456-s001.zip › toxics-2366107-supplementary.pdf]

Supplementary materials (S1)

Mercury biogeochemical cycle in Yanwuping Hg mine and source apportionment by Hg isotopes

Xingang Jin [1], Junyao Yan [2], Muhammad Ubaid Ali [2], Qiuhua Li [1,*] and Ping Li [2,*]

[1]Key Laboratory for Information System of Mountainous Area and Protection of Ecological Environment of Guizhou Province, Guizhou Normal University, Guiyang 550001, China; jinxingang0725@foxmail.com

[2] State Key Laboratory of Environmental Geochemistry, Institute of Geochemistry, Chinese Academy of Sciences, Guiyang 550081, China; yanjunyao@mail.gyig.ac.cn (J.Y.); ubaid@mail.gyig.ac.cn (M.U.A.)

*Correspondence: qiuhua2002@126.com (Q. L.); liping@mail.gyig.ac.cn (P. L.)

Supplementary Information

1. Supplementary figures

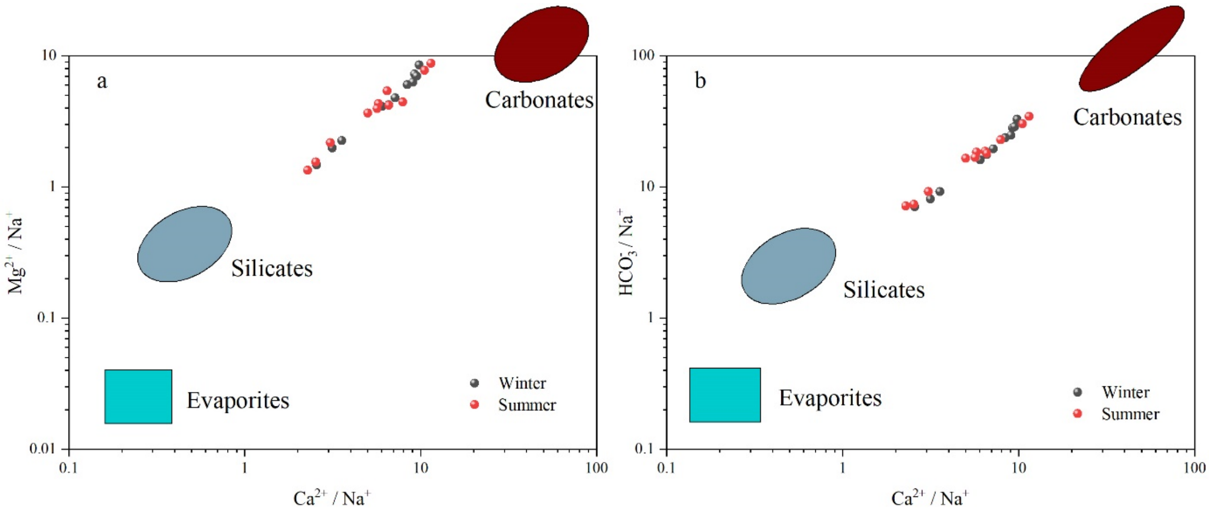

Figure S1. Molar ratio bivariate plots of (a) Na^+-normalized Ca^{2+} and Mg^{2+} and (b) Na^+-normalized Ca^{2+} and HCO_3^-. Rock weathering endmembers are cited from Gaillardet et al. [1,2].

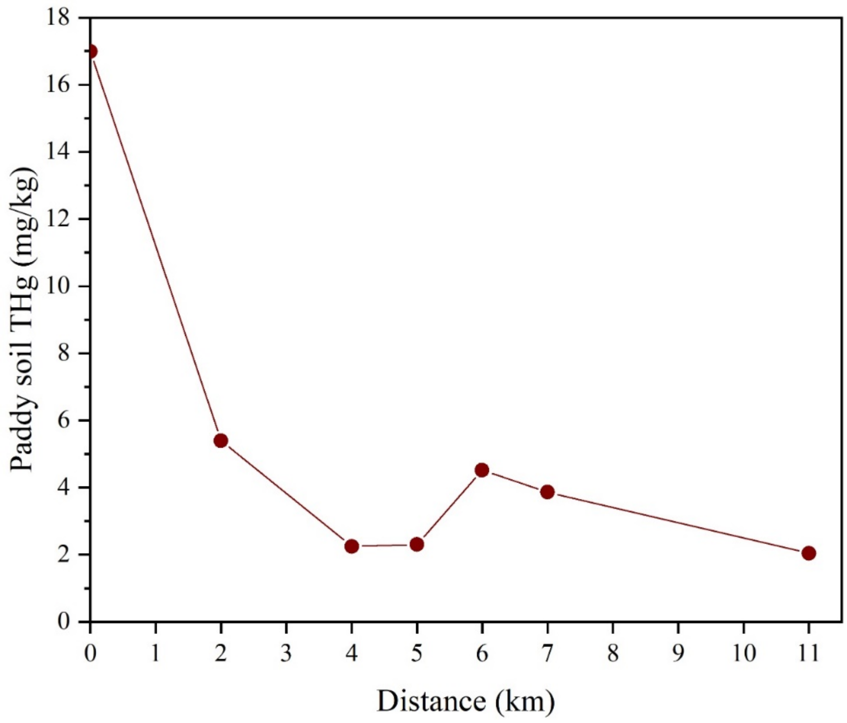

Figure S2. Variation of THg with distance in paddy field downstream of YMM.

2. Supplementary tables

Table S1. Mercury isotopes composition of water samples.

Sample ID	THg (ng/L)	$\delta^{202}Hg$ (‰)		$\Delta^{199}Hg$ (‰)		$\Delta^{201}Hg$ (‰)	
		Mean	SD	Mean	SD	Mean	SD
River water 1 DHg	37.2	-1.25	0.05	0.15	0.04	0.02	0.04
River water 1 PHg	1.22×10^3	-0.48	0.05	-0.02	0.04	-0.07	0.04
River water 2	24.2	-0.71	0.05	0.04	0.04	-0.06	0.04
River water 4	10.3	-0.14	0.05	-0.12	0.04	-0.26	0.04
River water 5	35.7	0.11	0.05	-0.08	0.04	-0.10	0.04
River water 6	31.3	-0.56	0.05	0.06	0.04	0.00	0.04
River water 7	9.55	-0.22	0.05	-0.05	0.04	-0.03	0.04
River water 11	23.4	-0.22	0.05	0.04	0.04	-0.05	0.04
Rain water 1	63.3	-0.51	0.05	-0.10	0.04	-0.09	0.04
Rain water 2	44.0	-0.34	0.05	0.30	0.04	0.19	0.04
Mountain spring water DHg	6.61	-1.57	0.05	0.15	0.04	0.04	0.04
Mountain spring water PHg	0.14	-1.78	0.05	-0.03	0.04	0.02	0.04

Table S2. Mercury isotopic composition in soil samples.

Sample ID	THg	$\delta^{202}Hg$ (‰)		$\Delta^{199}Hg$ (‰)		$\Delta^{201}Hg$ (‰)	
	(mg/kg)	Mean	SD	Mean	SD	Mean	SD
Paddy soil 1-1	2.27	-0.56	0.05	0.01	0.04	-0.01	0.04
Paddy soil 1-2	8.51	-0.91	0.05	0.04	0.04	0.00	0.04
Paddy soil 2	2.31	-0.82	0.05	-0.05	0.04	-0.05	0.04
Paddy soil 3-1	1.49	-0.64	0.05	0.07	0.04	0.02	0.04
Paddy soil 3-2	3.29	-0.80	0.05	0.08	0.04	0.09	0.04
Paddy soil 3-3	2.13	-0.72	0.05	0.1	0.04	0.06	0.04
Paddy soil 4-2	4.96	-0.67	0.05	0.02	0.04	-0.11	0.04
Paddy soil 4-3	4.01	-0.90	0.06	0.04	0.03	-0.03	0.02
Paddy soil 5-1	4.55	-0.73	0.05	0.08	0.04	0.06	0.04
Paddy soil 5-2	2.29	-0.58	0.05	0.05	0.04	-0.01	0.04
Paddy soil 5-3	4.92	-0.73	0.05	-0.04	0.04	-0.03	0.04
Paddy soil 6	2.04	-0.74	0.05	-0.01	0.04	-0.05	0.04
Natural background soil 1*	1.94	-1.30	0.06	0.00	0.08	-0.18	0.04
Natural background soil 2*	3.17	-1.21	0.04	-0.14	0.02	-0.08	0.02
Total soluble Hg	0.03	-0.90	0.05	0.06	0.04	0.05	0.04
Calcines	43.8	-0.35	0.05	-0.03	0.04	-0.08	0.04
CC580	131	-0.47	0.04	-0.06	0.02	-0.04	0.02
UM-Almadén standard solution		-0.52	0.05	0.00	0.04	-0.02	0.04

* Cited from Song et al. [3].

References

1. Gaillardet, J.; Dupre, B.; Allegre, C.J.; Negrel, P. Chemical and physical denudation in the Amazon River basin. *Chemical Geology* **1997**, 142, 141-173.

2. Gaillardet, J.; Dupre, B.; Louvat, P.; Allegre, C.J. Global silicate weathering and CO_2 consumption rates deduced from the chemistry of large rivers. *Chemical Geology* **1999**, 159, 3-30.

3. Song, Z.; Wang, C.; Ding, L.; Chen, M.; Hu, Y.; Li, P.; Zhang, L.; Feng, X. Soil mercury pollution caused by typical anthropogenic sources in China: Evidence from stable mercury isotope measurement and receptor model analysis. *Journal of Cleaner Production* **2021**, 288, 125687.
